# Down-regulation of the PI3K/Akt signaling pathway and induction of apoptosis in CA46 Burkitt lymphoma cells by baicalin

**DOI:** 10.1186/1756-9966-31-48

**Published:** 2012-05-20

**Authors:** Yi Huang, Jianda Hu, Jing Zheng, Jing Li, Tiannan Wei, Zhihong Zheng, Yingyu Chen

**Affiliations:** 1 Fujian Institute of Hematology, Fujian Provincial Key Laboratory of Hematology, Fujian Medical University Union Hospital, 29 Xinquan Road, Fuzhou, 350000, Fujian, China; 2 Provincial Clinical College of Fujian Medical University, 134 Dong Street, Fuzhou, 350000, Fujian, China

**Keywords:** Apoptosis, Intrinsic pathway, Baicalin, Burkitt lymphoma, CA46 cells, PI3K/Akt signaling pathway

## Abstract

**Background:**

Baicalin, a flavone present in *Scutellaria baicalensis Georgi*, inhibits the growth of human leukemia and myeloma cells through induction of apoptosis.

**Methods:**

The present study was undertaken to ascertain whether cultured Burkitt lymphoma cells undergo apoptosis when treated with baicalin. Growth rates were measured using MTT and colony formation assays, and induction of apoptosis was quantified using Annexin V and DNA fragmentation assays. Mechanisms underlying observed growth suppression were examined using Western blotting.

**Results:**

Treatment of CA46 Burkitt lymphoma cells with baicalin for 48 h markedly decreased the rate of cell proliferation; an IC_50_ value of 10 μM was obtained. Colony formation was almost fully suppressed at 10 μM baicalin. CA46 cells underwent apoptosis in response to baicalin treatment as evidenced by an increase in the percentage of cells stainable with Annexin V, by increased DNA fragmentation, and by activation of the intrinsic (mitochondrial) pathway for cell death as characterized by increased expression of the cleaved forms of caspase-9, caspase-3, and poly (ADP-ribose) polymerase. Additionally, baicalin was found to down-regulate anti-apoptotic and up-regulate apoptotic components of the phosphatidylinositide-3-kinase (PI3K)/serine/threonine kinase (Akt) signaling pathway.

**Conclusions:**

The concentrations at which baicalin altered expression of components of the PI3K/Akt pathway in CA46 cells were comparable to those that suppressed growth and induced apoptosis, supporting the hypothesis that the observed growth-inhibitory and apoptosis-inducing actions of baicalin in these cells are mediated by down-regulation of this pathway.

## Introduction

Burkitt lymphoma is a high-grade, rapidly-growing and aggressive B-cell non-Hodgkin's lymphoma [1]. Three forms are recognized: those endemic to Africa, sporadic forms, and those associated with immunodeficiency states. In the endemic and sporadic forms, B lymphocytes possess rearranged immunoglobulin genes and most commonly carry the (8;14) chromosomal translocation of the proto-oncogene *c-myc*[1]. Although Burkitt lymphoma is sensitive to chemotherapy, the different regimens used to treat this cancer are associated with varied success rates [1,2]. Prognosis depends on the stage of the disease at diagnosis and is generally worse for children, adolescents, and patients with co-existent AIDS.

Baicalin is one of several pharmacologically-active flavones present in *Scutellaria baicalensis Georgi* (Huang-qin or Chinese skullcap), a plant widely used in traditional Chinese herbal medicine [3]. Although baicalin is generally non-toxic to normal tissues, it exhibits strong anti-inflammatory, anti-viral, and anti-tumor activities [4,5]. Growth of human leukemia and myeloma cells and of human hepatic, prostate, breast, lung, bladder, and estrogenic cancer cells is potently suppressed by this flavone. Molecular mechanisms underlying these growth-suppressive effects are thought to include changes in oxidation/reduction status, cell cycle inhibition, and induction of apoptosis [3-5]. Baicalin-induced apoptosis in human leukemia cell lines is mediated by production of reactive oxygen species, induction of growth arrest and DNA damage-inducible protein 153 (GADD153), decreased expression of the anti-apoptotic protein Bcl-2, activation of the intrinsic (mitochondrial) apoptotic pathway, DNA fragmentation, and cycle arrest at the G0/G1 boundary [6,7]. Treatment of doxorubicin-resistant human myeloid leukemia cells with baicalin results in decreased expression of Bcl-2, c-myc, procaspase-3, and poly(ADP-ribose) polymerase (PARP), increased expression of Bad and cleaved PARP, and enhanced sensitivity to doxorubicin [8]. The growth of certain types of cultured lymphoma cells has been found to be suppressed by treatment with *Scutellaria baicalensis* extracts containing 21% baicalin [9]. However, no studies that examine the effects of baicalin on lymphoma cell proliferation have been reported.

The phosphatidylinositol-3-kinase (PI3K)/serine/threonine kinase (Akt) signaling pathway is essential to the survival and proliferation of human cells, and constitutive activation of this pathway is thought to play a critical role in the progression of human hematologic malignancies [10,11]. Inhibitors of this pathway have been shown to induce apoptosis in isolated leukemia, lymphoma, and myeloma cells. The CA46 lymphoma cell line [12], which was derived from the ascites fluid of a patient with American-type Burkitt lymphoma, carries the (8;14) translocation, overexpresses *Bcl-2* and *c-myc* mRNAs, and has been proven a useful model of Burkitt lymphoma. The following study was undertaken to ascertain whether baicalin down-regulates the PI3K/Akt signaling pathway in CA46 cells concurrently with induction of apoptotic cell death.

## Materials and methods

### Materials

Baicalin (C_21_H_18_O_11_, MW 446.35) was purchased from Qingzhe (Nanjing, Jiangsu, China). A 50 mM stock solution was prepared by dissolving 22.3 mg of the drug in 1 ml of dimethyl sulfoxide (DMSO; Sigma, St. Louis, MO, USA). The stock solution was maintained at −20°C and was diluted to appropriate concentrations with culture medium immediately before experimental use. Under these conditions, no baicalin solubility issues were encountered. The highest final concentration of DMSO in baicalin-treated preparations was 0.08%; the viability of control preparations was unaffected at this DMSO concentration.

### Cell culture

The Jurkat, K562, HL-60, and CA46 Burkitt lymphoma cell lines were obtained from the China Center for Type Culture Collection (CCTCC; Wuhan, Hubei, China). Cultures were maintained in RPMI-1640 medium (Gibco, Grand Island, NY, USA) with 10% fetal bovine serum (FBS; Gibco, Grand Island, NY, USA) at 37°C in a humidified atmosphere containing 5% CO_2_.

### Proliferation assay

The 3-(4,5-dimethylthiazol-2-yl)-2,5 diphenyltetrazolium bromide (MTT) assay was used to measure the rate of cell proliferation. Briefly, CA46 cells (1 × 10^4^/well) were seeded in 96-well plates and treated with baicalin at varying concentrations. After varying incubation times, cells were treated with 20 μl of MTT solution (Sigma, St. Louis, MO, USA) at a final concentration of 5 mg/ml for 4 h at 37°C. Medium was then removed, DMSO (200 μl) was added, and the absorbance maxima at test and reference wavelengths of 490 and 630 nm, respectively, were recorded. The proliferation inhibitory rate (%) was calculated as: [1-(absorbance of baicalin treated group/absorbance of control group)] × 100.

### Colony-forming assay

CA46 cells were seeded at a density of 4 × 10^2^/well in 24-well flat bottom plates and then cultured with baicalin at different concentrations in RPMI-1640 medium with 10% FBS and 0.7% methylcellulose at 37°C for 10 days. Colony formation was observed using phase contrast inverse microscopy. The resulting cell colonies (>50 cells/colony) were counted, and colony formation rate (%) was calculated as: (formed colonies/seeded cells) × 100.

### Measurements of cells in early and late apoptosis

The ability of baicalin to induce apoptosis in CA46 cells was examined by Annexin V-FITC/PI double-staining and flow cytometry. Preparations were treated with baicalin at varying concentrations for 48 h. Cells were then harvested, resuspended to 5 × 10^5^ /ml in binding buffer (HEPES, 10 mM, pH 7.4, 150 mM NaCl, 5 mM KCl, 1 mM MgCl_2_, 1.8 mM CaCl_2_), and doubly stained with Annexin V-Fluorescein Isothiocyanate (FITC)/Propidium Iodide (PI) (BD, Franklin, NJ, USA) according to the manufacturer’s instructions. The percentages of viable, early apoptotic, late apoptotic, and necrotic cells were determined using a CPICX XL flow cytometer (Beckman Coulter, Fullerton, CA, USA).

### DNA fragmentation assay

After 48 h exposure to baicalin at varying concentrations, CA46 cells were collected by centrifugation and washed twice with PBS. Cell pellets were resuspended in 40 μl of lysis buffer (0.1 M EDTA, 0.1 M Tris–HCl pH 8.0, 0.8% SDS) and subsequently treated with 10 μl RNase A (50 μg/ml) at 37°C for 1 h and with 10 μl proteinase K (20 μg/ml) at 50°C overnight. Extracted cellular DNA was subjected to agarose gel (2.0%) chromatography at 35 V for 3 h. Gels were photographed after staining with 0.5 μg/ml ethidium bromide.

### Western blot analyses

Western blotting was performed as described previously [8]. CA46 cells were treated with 40 μM baicalin for 0–72 h prior to lysis. Protein Detector LumiGLO Western Blot Kits were purchased from KPL (Gaithersburg, MD, USA). Antibodies to the following proteins were used for these analyses: β-actin (NeoMarkers, Fremont, CA, USA); Akt, p-Akt (Ser473), mammalian target of rapamycin (mTOR), p-mTOR (Ser2448), IκB, p-IκB (Ser 32), PARP, cleaved caspase-9 (Asp330), and cleaved caspase-3 (Asp175) (Cell Signaling, Danvers, MA, USA); NF-κB p65 (eBioscience, San Diego, CA, USA). The density of β-actin served as an internal loading control.

### Statistical analysis

Experimental findings are expressed as means ± standard deviation. Comparisons involving different baicalin concentrations or incubation times were conducted using analysis of variance (ANOVA). Multiple comparisons were performed using the Bonferroni procedure with type-I error adjustment when significance was obtained. The level of significance was set at 0.05. Statistical analyses were performed using SAS 9.1 statistical software (SAS Institute Inc., Cary, NC, USA).

## Results

### Inhibition of cell proliferation and colony formation

**Figure 1:**
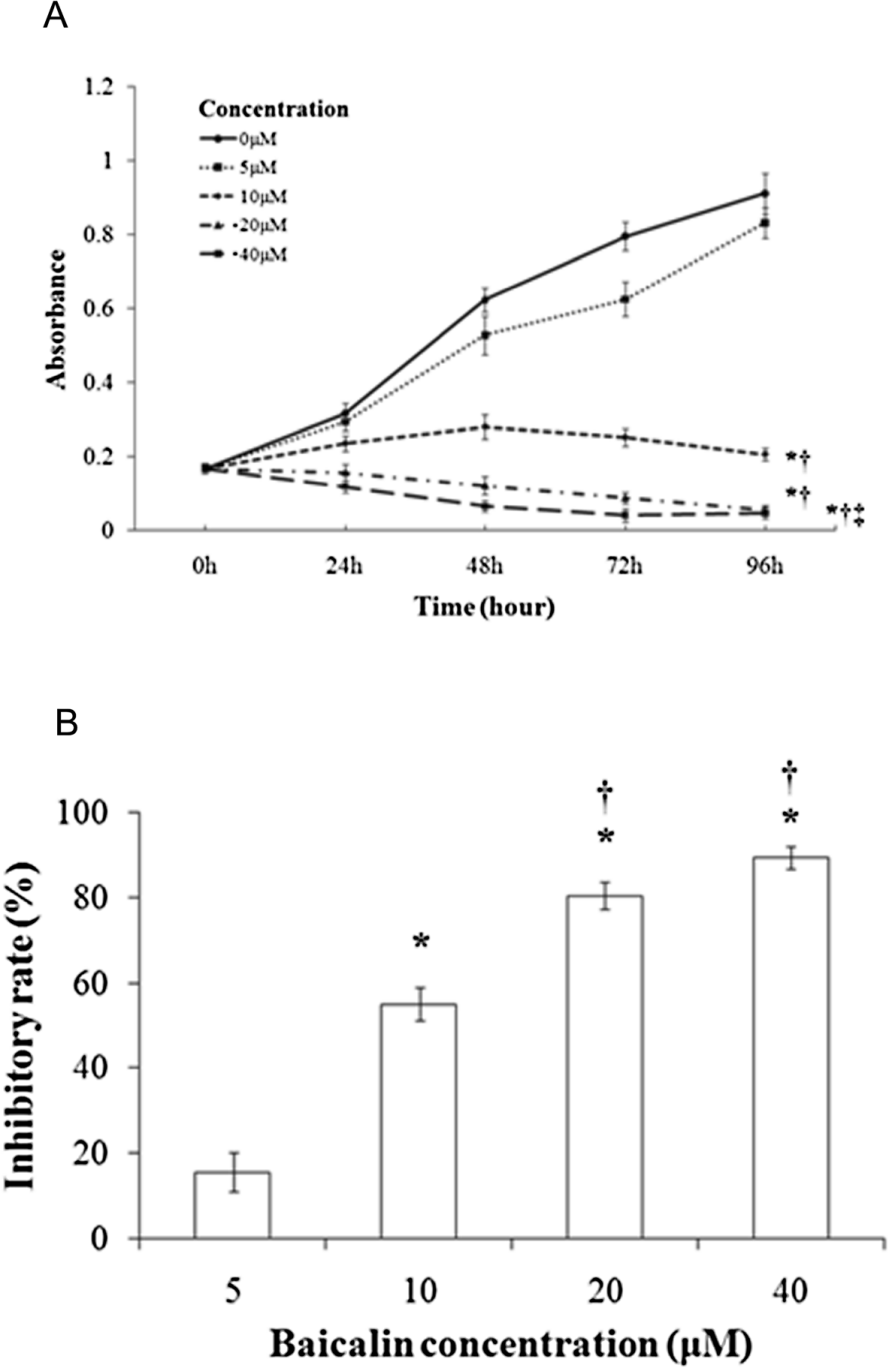
**Proliferation of CA46 cells in the absence and presence of baicalin.** Cells were seeded at a density of 1 × 10^4^/well and treated with baicalin at the concentrations and for the times indicated. Cytotoxicity was determined according to the MTT assay. Sampling was performed in triplicate for each experimental condition, and findings are expressed as means ± standard deviation for three independent experiments. **(A)** Proliferation as a function of incubation time and baicalin concentration. Absorbance maxima are provided on the ordinate. **(B)** Rates of proliferation as a function of baicalin concentration. Cells were treated for 48 h with baicalin at the concentrations indicated. Proliferation rates were determined as described in Materials and methods. *P <0.01 compared to the solvent control; ^†^*P* <0.01 compared to 5 μM baicalin; ^‡^*P* <0.01 compared to 10 μM baicalin.

**Figure 2:**
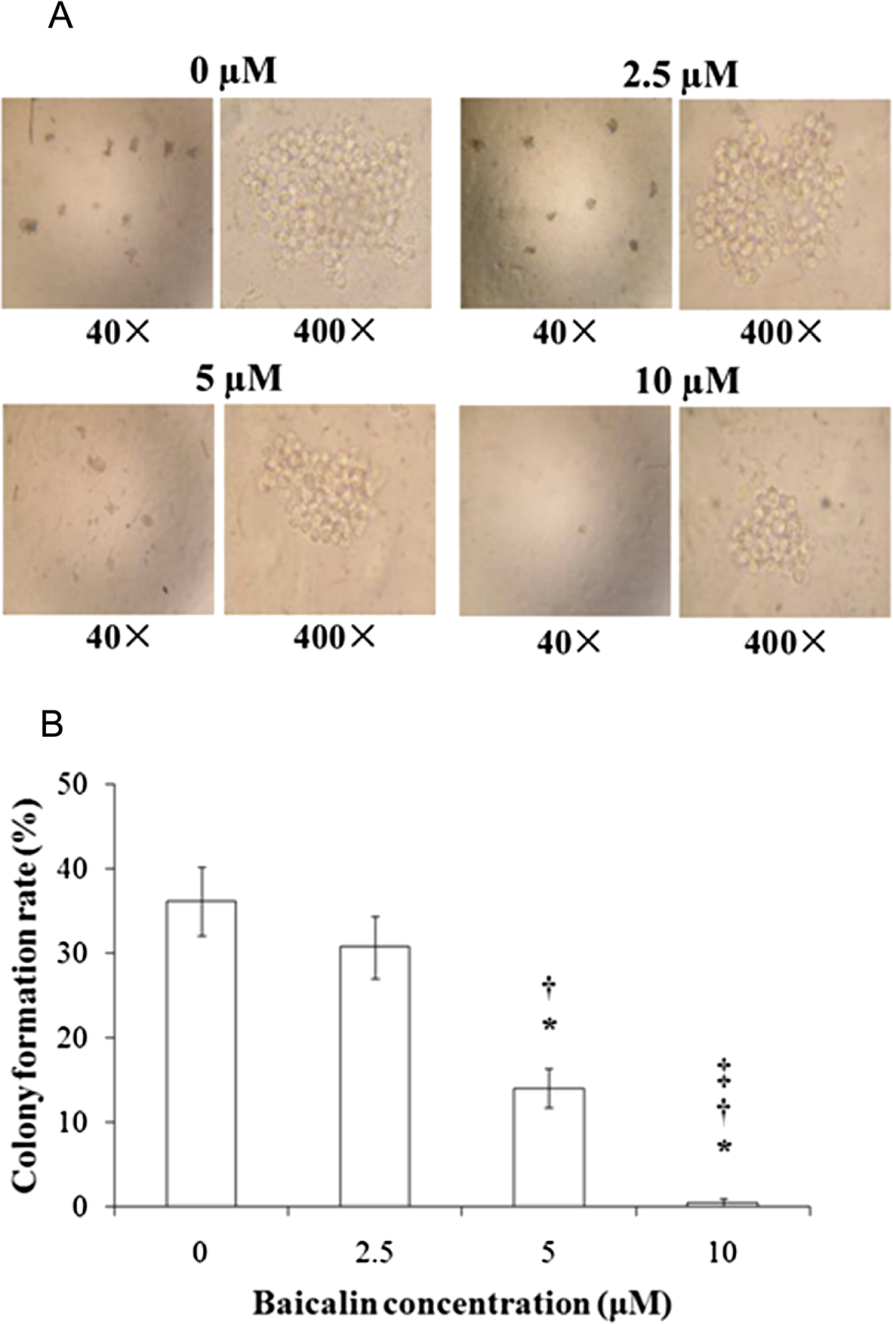
**Formation of CA46 cell colonies after treatment with baicalin at varying concentrations.** Cells (4 × 10^2^/well) were cultured with baicalin at the indicated concentrations for 10 days. Colony formation rates were determined as described in Materials and methods. Sampling was performed in triplicate for each experimental condition. **(A)**, phase contrast inverse microscopy. **(B)**, colony formation rates with findings presented as means ± standard deviation for three independent experiments. *P <0.01 compared to the solvent control; ^†^*P* <0.01 compared to 2.5 μM baicalin; ^‡^*P* <0.01 compared to 5 μM baicalin.

Baicalin inhibited the proliferation of CA46 cells in a concentration- and time-dependent manner, with almost complete inhibition observed at 48–96 h of treatment with 20–40 μM drug (Figure 1A). An IC_50_ of 10 μM was obtained (Figure 1B). After 48 h of treatment, rates of proliferation declined in a baicalin concentration-dependent manner, with 15.5 ± 4.7% and 89.4 ± 2.8% inhibitions observed at 5 and 40 μM drug, respectively. Baicalin also suppressed formation of colonies of CA46 cells at 10 days post-seeding (Figures 2A and 2B). Control preparations formed colonies at a rate of 36.2 ± 4.0%. In contrast, rates of colony formation for preparations treated with baicalin at 5 and 10 μM were 14.0 ± 2.3% and 0.5 ± 0.5%, respectively (*P* <0.01).

### Induction of apoptosis

**Figure 3:**
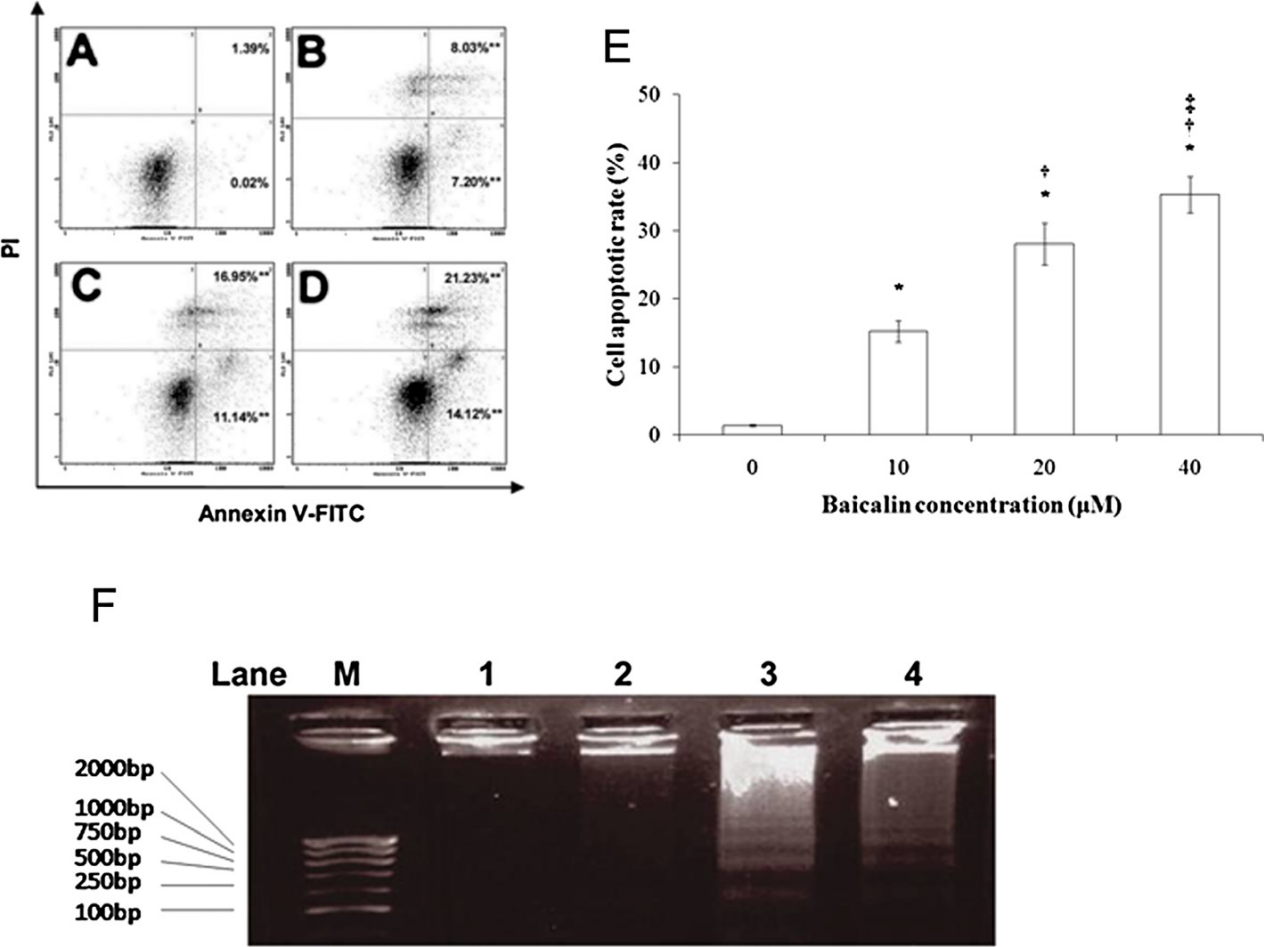
**Induction of apoptosis in CA46 cells by baicalin.** Annexin V-FITC/PI double staining and flow cytometry were used to determine the percentages of cells in apoptosis. Viable, early apoptotic, late apoptotic, and necrotic cells were determined after 48 h treatments with baicalin at varying concentrations. Cells were treated with baicalin at **(A)** 0, **(B)** 10, **(C)** 20, and **(D)** 40 μM. Bottom left quadrants, viable cells; bottom right quadrants, early apoptotic cells; top right quadrants, late apoptotic cells; top left quadrants, necrotic cells. **(E)** Percentages of cells in apoptosis at each baicalin concentration. Cells in the bottom right and top right quadrants were summed to obtain the percentage of all cells in apoptosis. Findings are presented as the means of three similar experiments ± standard deviation. **(F)** CA46 cells were treated for 48 h with baicalin at 0 (lane 1), 10 (lane 2), 20 (lane 3), and 40 (lane 4) μM. Cellular DNA was extracted and subjected to agarose gel electrophoresis as described in Materials and methods. Gels were stained with ethidium bromide and photographed. Lane M presents migration of D2000-Markers (100, 250, 500, 750, 1000, 2000 bp). Findings are representative of those obtained on three separate occasions. *P <0.05 compared to the solvent control; ^†^*P* <0.05 compared to 10 μM baicalin; ^‡^*P* <0.05 compared to 20 μM baicalin.

The percentage of CA46 cells undergoing apoptotic cell death was increased by baicalin in a concentration-dependent manner (Figure 3A-D). The percentages of all cells in apoptosis, as determined by the sum of cells in early and late apoptosis, at various baicalin concentrations are presented in Figure 3E. After 48 h of treatment, 15.2 ± 1.6% of cells were apoptotic at 10 μM baicalin and 35.4 ± 2.6% of cells were apoptotic at 40 μM baicalin. When the ability of baicalin to induce DNA fragmentation, a hallmark of apoptosis, was examined after 48 h of culture (Figure 3F), no significant fragmentation was observed in preparations treated with solvent or 5 μM baicalin. However, fragmentation was clearly observable in preparations treated with 20 and 40 μM baicalin.

### Suppression of the PI3K/Akt pathway

**Figure 4:**
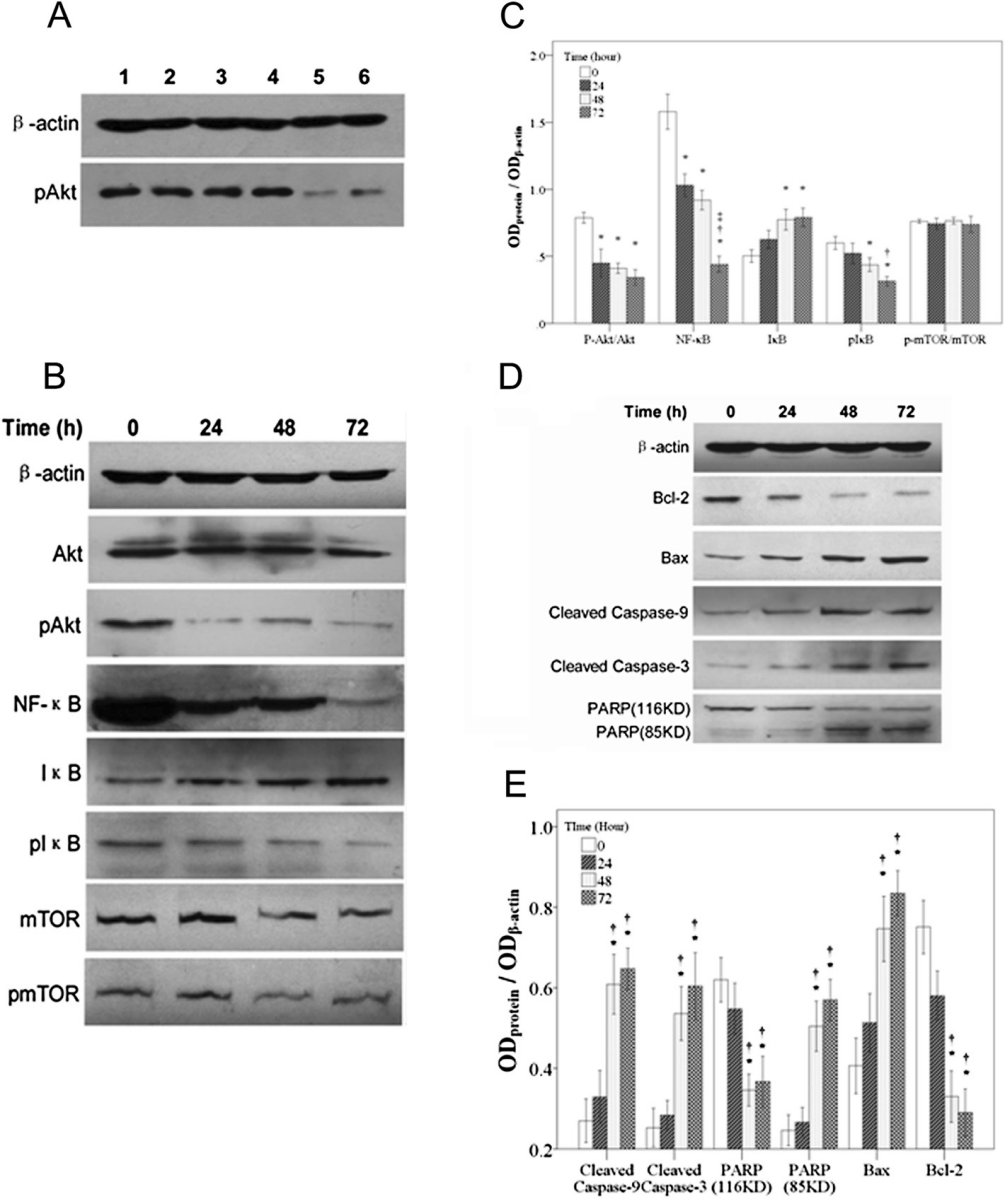
**Expression of proteins associated with the PI3K/Akt signaling and the intrinsic (mitochondrial) apoptotic pathways after varying times of treatment of CA46 cells with baicalin.(A)** Expression of p-Akt in various untreated cell types as detected by phospho-Akt specific antibody. Lane 1, CA46 cells; lane 2, Jurkat cells; lane 3, K562 cells; lane 4, HL-60 cells; lane 5, normal peripheral blood mononuclear cells-1; lane 6, normal peripheral blood mononuclear cells-2. **(B-E)** CA46 cells were treated with 40 μM baicalin for the times indicated. Protein expression was analyzed by Western blotting. **(B)** Western blot showing expression of β-actin, Akt, p-Akt, NF-κB, IκB, p-IκB, mTOR and p-mTOR. **(C)** Expression of p-Akt/Akt, NF-κB, IκB, p-IκB, and p-mTOR/mTOR relative to that of β-actin. **(D)** Western blot showing expression of β-actin, Bcl-2, Bax, cleaved caspase-9, cleaved caspase-3, and uncleaved (116 kD) and cleaved (85 kD) PARP. **(E)** Expression of cleaved caspase-9, cleaved caspase-3, uncleaved and cleaved PARP, Bax, and Bcl-2 relative to that of β-actin. Findings are representative of those obtained on three separate occasions. *P <0.05 compared to the 0 h control; ^†^*P* <0.05 compared to 24 h treatment; ^‡^*P* <0.05 compared to 48 h treatment.

The possibility that the induction of apoptosis in CA46 cells by baicalin involved suppression of Akt signaling was explored. Basal expression of p-Akt (the activated form of Akt) was examined in C46 cells, in three leukemic cell types, and in normal peripheral blood mononuclear cells under untreated conditions. As compared to normal peripheral blood mononuclear cells, high degrees of p-Akt expression were observed in C46 lymphoma cells and in all types of leukemic cells (Figure 4A). The effects of baicalin on expression of Akt and of specific downstream components of the Akt pathway in CA46 cells were then examined. Expression of the following components in their various forms was measured: (a) Akt (inactive) and p-Akt; (b) the transcription factor NF-κB, the NF-κB inhibitor, IκB, and the degradable form of IκB, p-IκB; (c) the cell cycle regulatory kinase mTOR (inactive) and p-mTOR, the phosphorylated and active form of the kinase. An increase in the dephosphorylated form of Akt was observed at 24 h of baicalin treatment, and an increase in the dephosphorylated form of mTOR was observed at 48 h of baicalin treatment. Dramatic reductions in expression of NF-κB and p-IκB were observed in response to baicalin; these reductions were time-dependent. By contrast, expression of IκB increased with time of baicalin treatment (Figure 4B). Changes in expression of these components were quantified, and the findings are summarized in Figure 4C.

The profound decreases in expression of total cellular NF-kB and p-IkB, accompanied by significant increases in IkB expression, in response to baicalin treatment were interpreted to indicate a condition wherein nuclear NF-kB signaling should be dramatically impaired. Accordingly, expression of nuclear NF-kB was reduced by 25.8%, 50.4% and 65.4% at 24, 48 and 72 h of treatment with 40 μM baicalin, respectively (not shown).

### Activation of the intrinsic mitochondrial apoptotic pathway

It was considered essential to ascertain whether baicalin suppresses proliferation of CA46 cells and promotes DNA fragmentation in these cells through activation of the intrinsic (mitochondrial) apoptotic pathway. To this end, expression of relevant apoptosis-related proteins was examined by Western blotting. Treatment with baicalin increased expression of the pro-apoptotic proteins Bax, activated (cleaved) caspase 3, activated (cleaved) caspase 9, and activated (cleaved) PARP. By contrast, expression of the anti-apoptotic protein Bcl-2 and of the inactive form of PARP was decreased following treatment with the drug (Figure 4D). Relative expression of these proteins after baicalin treatment was quantified, and findings are presented in Figure 4E. The concentrations at which baicalin altered expression of these apoptosis-related proteins were similar to those at which cell proliferation was suppressed and expression of components of the PI3K/Akt signaling pathway was altered, supporting the hypothesis that the growth-inhibitory and apoptosis-inducing actions of baicalin in CA46 cells are mediated by suppression of PI3K/Akt signaling pathway.

## Discussion

The present study is the first to demonstrate that baicalin is toxic to Burkitt lymphoma cells in culture. Treatment with this flavone at 10 μM concentrations resulted in a marked decrease in the rate of proliferation of cultured CA46 cells and in the rate at which these cells formed colonies. Baicalin treatment caused CA46 cells to undergo apoptosis as evidenced by an increase in the percentage of Annexin V-stainable cells and by increased DNA fragmentation. Baicalin also activated the mitochondrial pathway for cell death, as shown by increased expression of activated caspase-9, activated caspase-3, and cleaved PARP. Treatment of CA46 cells with baicalin was found to suppress components of the PI3K/Akt signaling pathway, as shown by decreased expression of p-Akt, mTOR, p-mTOR, NF-κB, and p-IκB. These decreases were observed concurrently with increased expression of non-phosphorylated IκB. The concentrations at which baicalin altered the expression of components of the PI3K/Akt signaling pathway were similar to those at which the drug suppressed growth and induced apoptosis, supporting the hypothesis that the growth-inhibitory and apoptosis-inducing actions of baicalin in CA46 cells are mediated by suppression of this pathway.

Although baicalin has been found to induce apoptosis in several malignant hematologic cell types, the mechanism responsible for the induction has not been examined in detail. Baicalin treatment has been shown to promote activation of the mitochondrial pathway of apoptosis and to induce DNA fragmentation and cycle arrest in human leukemia cells but the upstream mechanisms responsible for these actions were not examined [6-8]. Baicalein, a non-glycosylated derivative of baicalin and one of the major flavones present in *Scutellaria baicalensis Georgi*, was recently reported to induce apoptosis in human myeloma cells through inhibition of Akt activation [13]. However, baicalein and baicalin are not identical in their cellular actions. Although both flavones induce apoptosis in several types of murine and human cancer cells, events mediating growth suppression by baicalein do not routinely duplicate those mediated by baicalin [14-19]. In addition, baicalin is unable to duplicate the baicalein-induced activation of the IL-6-mediated signaling cascade seen in human myeloma cells [13]. Whether baicalein is similar to baicalin in its action on Akt and downstream mediators in Burkitt lymphoma cells remains to be demonstrated.

The PI3K/Akt growth signaling pathway is comprised of a family of intracellular protein kinases, each of which is regulated by phosphorylation and possesses unique substrate specificity. Activated Akt, the primary mediator of PI3K-initiated signaling, supports survival of various hematologic malignancies through its ability to phosphorylate and activate a wide variety of downstream targets [10,20,21]. In the present study, inhibition of Akt phosphorylation, rather than down-regulation of Akt expression, was observed during treatment of CA46 cells with baicalin. Since Akt is an early player in the PI3K/Akt signaling pathway, it is conceivable that the growth-suppressive effects of baicalin in CA46 cells are attributable to an interaction of the drug with the kinase. In support of this hypothesis, selective inactivation of Akt in Jurkat T lymphoblastic leukemia cells causes these cells to undergo apoptotic death via the mitochondrial pathway [22]. Because PI3K expression/activity was not measured in the present study, the involvement of this kinase in the observed effects of baicalin remains unclear. Future studies with various lymphoma cells lines are planned to explore the possibility that PI3K is targeted by baicalin.

NF-κB and mTOR, downstream components of the PI3K/Akt pathway, are thought to function importantly in maintenance of hematologic malignancies [10,11,20,23-25]. The transcription factor NF-κB is inactivated when complexed with IκB in the cytosol. Phosphorylation of IκB renders it a substrate for degradation, resulting in translocation of free NF-κB to the nucleus and transcriptional activation of anti-apoptotic genes. Activated Akt indirectly signals IκB phosphorylation, thereby promoting transcription of anti-apoptotic genes, whereas inactivation of Akt promotes apoptosis. mTOR is directly phosphorylated by activated Akt. Phosphorylated mTOR, the active form of the kinase, promotes cell cycle transition from the G1 to S phase via phosphorylation of its two downstream targets, p70 S6 kinase and eukaryotic initiation factor 4E-binding protein 1. These phosphorylations favor translation of mRNAs for certain growth-promoting proteins such as cyclin D and c-myc. Accordingly, pharmacologic antagonists of mTOR are anticipated to be effective against many types of solid tumors and hematologic cancers [10,11,25]. In the present study, expression of NF-κB, p-IκB, mTOR, and p-mTOR was found to be down-regulated in baicalin-treated CA46 cells. These findings support the hypothesis that induction of apoptosis in CA46 cells by baicalin is mediated by the suppression of PI3K/Akt/NF-κB and PI3K/Akt/mTOR signaling.

Suppression of Akt in cancer cells is associated with activation of the mitochondrial apoptotic pathway involving the caspase-9-dependent caspase cascade [20,24]. Treatment of CA46 cells with baicalin was found to increase the level of cleaved caspase-9 concurrently with a decrease in procaspase-9 protein, to increase level of cleaved caspase-3 concurrently with a decrease in procaspase-3 protein, to increase expression of cleaved PARP concurrently with decreased expression of uncleaved PARP, and to promote DNA degradation. These findings support the proposal that apoptotic death in baicalin-treated CA46 cells is mediated by the following events in sequence: cleavage of procaspase-9, cleavage of procaspase-3, cleavage of PARP, and degradation of DNA.

Based on the findings presented in this report, it is suggested that baicalin induces apoptosis in CA46 Burkitt lymphoma cells through down-regulation of the PI3K/Akt signaling pathway and activation of the mitochondrial death pathway. The findings described in this report warrant further investigations of the efficacy of baicalin against this form of lymphoma.

### Support and Financial Disclosure Declaration

This work was supported by grants from National Science & Technology Pillar Program (2008BAI61B01), the Fujian Bureau of Education (NCEFJ-0604), the Fujian Bureau of Public Health (2001-CX-02), and Fujian Medical University (JS06081).

## Competing interests

The authors declare that they have no competing interests.

## Authors’ contributions

YH: guarantor of integrity of the entire study, study concepts, study design, definition of intellectual content, literature research, experimental studies, data acquisition, data analysis, statistical analysis, manuscript preparation, manuscript editing, manuscript review, JH: guarantor of integrity of the entire study, study concepts, study design, definition of intellectual content, literature research, manuscript editing, manuscript review, JZ: experimental studies, data acquisition, JL: experimental studies, data acquisition, TW: data analysis, ZZ: statistical analysis, YC: manuscript preparation. All authors read and approved the final manuscript.
